# Protective Effects of Intratracheally-Administered Bee Venom Phospholipase A2 on Ovalbumin-Induced Allergic Asthma in Mice

**DOI:** 10.3390/toxins8100269

**Published:** 2016-09-22

**Authors:** Kyung-Hwa Jung, Hyunjung Baek, Dasom Shin, Gihyun Lee, Sangwon Park, Sujin Lee, Dabin Choi, Woojin Kim, Hyunsu Bae

**Affiliations:** Department of Physiology, College of Korean Medicine, Kyung Hee University, 26 Kyungheedae-ro, Dongdaemoon-gu, Seoul 130-701, Korea; jhkh242@naver.com (K.-H.J.); bguswjd@naver.com (H.B.); ssd060@naver.com (D.S.); jazz0705@hanmail.net (G.L.); genesispsw@naver.com (S.P.); su11024@naver.com (S.L.); dphs0228@naver.com (D.C.); wjkim@khu.ac.kr (W.K.)

**Keywords:** asthma, ovalbumin, bvPLA2, airway inflammation

## Abstract

Asthma is a common chronic disease characterized by bronchial inflammation, reversible airway obstruction, and airway hyperresponsiveness (AHR). Current therapeutic options for the management of asthma include inhaled corticosteroids and β2 agonists, which elicit harmful side effects. In the present study, we examined the capacity of phospholipase A2 (PLA2), one of the major components of bee venom (BV), to reduce airway inflammation and improve lung function in an experimental model of asthma. Allergic asthma was induced in female BALB/c mice by intraperitoneal administration of ovalbumin (OVA) on days 0 and 14, followed by intratracheal challenge with 1% OVA six times between days 22 and 30. The infiltration of immune cells, such as Th2 cytokines in the lungs, and the lung histology, were assessed in the OVA-challenged mice in the presence and absence of an intratracheal administration of bvPLA2. We showed that the intratracheal administration of bvPLA2 markedly suppressed the OVA-induced allergic airway inflammation by reducing AHR, overall area of inflammation, and goblet cell hyperplasia. Furthermore, the suppression was associated with a significant decrease in the production of Th2 cytokines, such as IL-4, IL-5, and IL-13, and a reduction in the number of total cells, including eosinophils, macrophages, and neutrophils in the airway.

## 1. Introduction

Allergic asthma is the most common chronic inflammatory disorder of the airway. The prevalence of asthma has significantly increased over the past few decades, particularly in children [1]. Asthma is characterized by airway and lung tissue inflammation and airway hyperresponsiveness (AHR) that leads to recurrent symptoms of wheezing, breathlessness, chest tightness, and coughing [1]. AHR indicates an exaggerated response of the airway to nonspecific stimuli, which results in a temporary airflow limitation, leading to airway obstruction. It remains unknown which factors within the airway of an individual trigger reversible airway obstruction and airway narrowing. The airway epithelium is composed of many interacting structural components and inflammatory cells. The number, activation, and secretory component of inflammatory cells in the airway are altered in the disease. In asthma, the number of eosinophils and T lymphocytes is increased in the subepithelial layer [2]. The number and activity of inflammatory cells is linked with AHR. Eosinophils predominate, and especially in asthmatics with nocturnal symptoms, probably reflect a more severe disease. Therapeutic drugs for the treatment of asthma include anti-allergic drugs, bronchodilators, and corticosteroids [3,4]. In an attempt to restrain the systemic delivery and the associated side effects, novel therapeutic drugs have been developed for direct administration into the lung. This approach offers an attractive solution for the treatment of respiratory diseases, such as inhaled corticosteroids and bronchodilators. It allows higher drug concentrations to be achieved at the target site with a lower systemic exposure than parenteral administration [5]. Bee venom, as an anti-inflammatory drug, has been used traditionally in the treatment of some immune-related disorders, such as rheumatoid arthritis and multiple sclerosis [6,7]. Phospholipase A2 is an enzyme that catalyzes the hydrolysis of the sn-2 fatty acyl bond of membrane phospholipids. Bee venom PLA2 (bvPLA2) is one of the major components of bee venom [8]. Our previous studies reported that bvPLA2 directly bound to the CD206 mannose receptor, which promoted immune tolerance through Treg cells modulation and prevented various inflammatory responses, such as neuroinflammation, airway inflammation, and acute kidney injury [9,10,11]. To study the impact of the route of administration on bvPLA2 penetration into the lungs, bvPLA2 was delivered by intratracheal instillation in an OVA-challenged allergic airway inflammation mouse model.

## 2. Results

### 2.1. Intratracheal Administration of bvPLA2 Alleviates Airway Hyperresponsiveness (AHR) to Methacholine Induced by OVA

**Figure 1:**
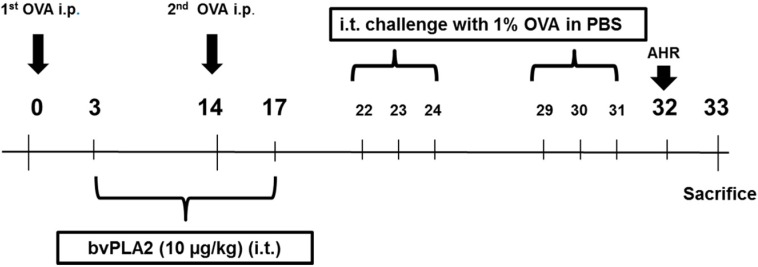
Experimental schedule. Seven-week-old female BALB/c mice were sensitized on days 0 and 14 by an intraperitoneal injection of OVA combined with 20 mg of aluminum hydroxide. From days 3–17, the mice were treated two times a week with PBS or bvPLA2 via i.t. injection, and then mice were challenged three times a week with an i.t. injection of OVA (1%) or PBS (days 22–31). Twenty-four hours after the OVA challenge, AHR was measured, and the mice were sacrificed on day 33.

**Figure 2:**
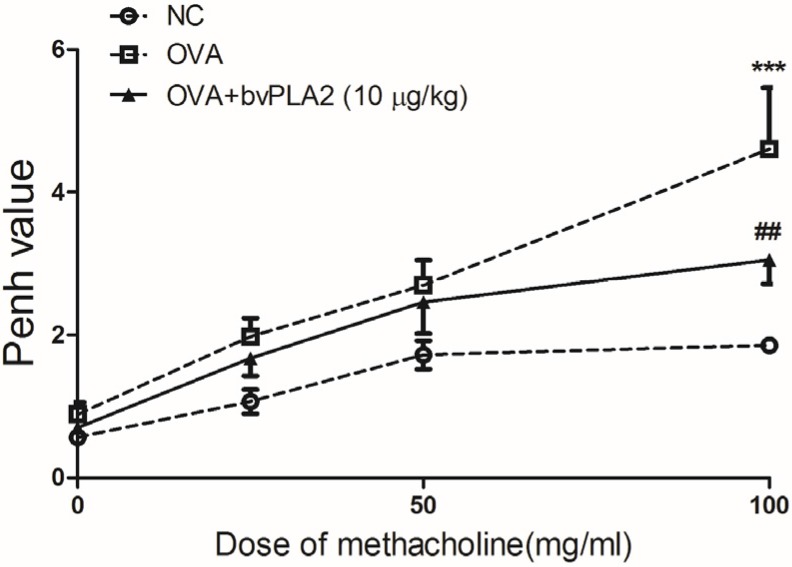
Effects of an intratracheal administration of bvPLA2 on airway AHR in OVA-challenged mice. Twenty-four hours after the final OVA or PBS challenge, the mice were measured for changes in AHR in response to different doses of methacholine in the NC, OVA, and OVA+PLA2 groups. All of the mice were stimulated with increasing doses of aerosolized methacholine (0, 25, 50 and 10 mg/mL). The normal control mice were treated with PBS alone (NC), the OVA challenged mice were treated with PBS (OVA) and the OVA challenged mice were i.t. treated with 10 µg/kg of bvPLA2 (OVA+PLA2 (10 µg/kg)). The statistical analyses were conducted by one-way ANOVA followed by Newman-Keuls multiple comparison test (*** *p* < 0.001 vs. NC group and ^##^ *p* < 0.01 vs. OVA+PLA2 (10 µg/kg) group; *n* = 5).

The mice were sensitized with OVA+alum, and challenged with OVA (1%) via intraperitoneal (i.p.) and intratracheal (i.t.) injection, respectively. On days 3–17, the sensitized mice were administered, intratracheally, bvPLA2 (10 µg/kg body wt) (Figure 1). To investigate whether the i.t. administration of bvPLA2 affects airway narrowing in an OVA-induced allergic airway inflammation model, the total respiratory airflow in mice was determined using a whole-body plethysmography. As expected, the Penh value of the OVA group was significantly increased compared with the NC group at 100 mg/mL of methacholine. Additionally, the Penh value was markedly decreased in the bvPLA2 treated group compared with the OVA group at 100 mg/mL of methacholine (Figure 2). Throughout the results, no statistically significant differences were found in the 0, 25, and 50 mg/mL of methacholine treatment in each group. These results demonstrate that the i.t. administration of bvPLA2 relieves AHR during allergic airway inflammation.

### 2.2. bvPAL2 Inhibits the Recruitment of Inflammatory Cells in BAL Fluid Induced by OVA

**Figure 3:**
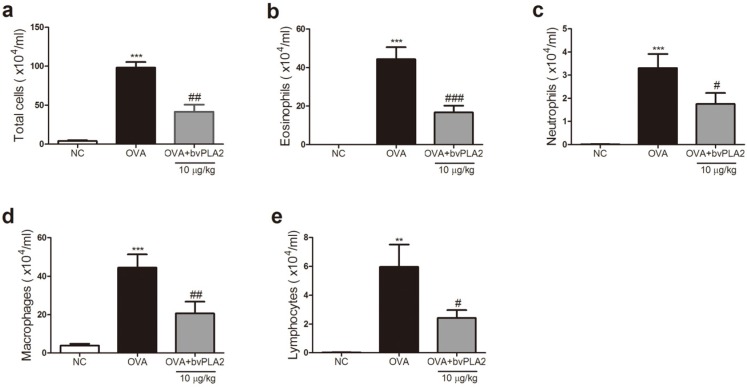
Effects of bvPLA2 on the influx of inflammatory cells in the BAL fluid of OVA-challenged mice. On day 33, the mice were sacrificed and BAL fluid was collected. The total cells and each inflammatory cell (eosinophils, neutrophils, macrophages and lymphocytes) were examined by counting a maximum of 500 cells on a smear prepared by using cytocentrifugation and Diff-Quick staining. The results are expressed as the number of each cell population in 1 mL of BAL fluid. The number of (**a**) total cells; (**b**) eosinophils; (**c**) neutrophils; (**d**) macrophages; and (**e**) lymphocytes. The statistical analyses were conducted by one-way ANOVA followed by Newman-Keuls multiple comparison test (*** *p* < 0.001, ** *p* <0.01 and * *p* <0.05 vs. NC group, ^###^ *p* < 0.001, ^##^ *p* < 0.01 and ^#^ *p* < 0.05 vs. OVA+PLA2 (10 µg/kg) group; *n* = 5).

To evaluate the inhibitory effect of the bvPLA2 on the influx of inflammatory cells, the immune cells were analyzed in the BAL fluid of OVA-induced allergic airway inflammation. Compared with PBS-treated mice, the OVA-challenged mice had significantly increased numbers of infiltrating total cells, including eosinophils, neutrophils, macrophages, and lymphocytes. These data indicated that ovalbumin exposure caused a noticeable influx of inflammatory cells in the BAL fluid. On the other hand, the bvPLA2-treated group showed a remarkable reduction in the number of total cells, such as eosinophils, neutrophils, macrophages, and lymphocytes, compared with the OVA group in the BAL fluid (Figure 3).

### 2.3. bvPLA2 Inhibits Th2 Cytokine Secretion in the Lungs Induced by OVA

**Figure 4:**
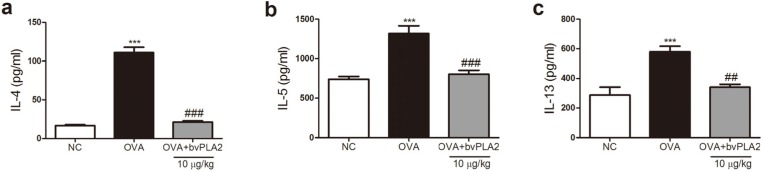
Effects of bvPLA2 on the secretion of Th2 cytokines in the lung tissues of OVA-challenged mice. The relative expression of IL-4, IL-5, and IL-13 were measured by ELISA. (**a**) Level of IL-4; (**b**) level of IL-5; and (**c**) level of IL-13. The statistical analyses were conducted by one-way ANOVA followed by Newman-Keuls multiple comparison test (*** *p* < 0.001 vs. NC group, ^###^ *p* < 0.001 and ^##^ *p* < 0.01 vs. OVA+PLA2 10 µg/kg group; *n* = 5).

To investigate the effects of bvPLA2 on OVA-induced Th2 cytokine expression levels in the lung tissues an enzyme-linked immunosorbent assay (ELISA) was performed. As shown in Figure 4, levels of IL-4, IL-5, and IL-13 significantly increased in the OVA group compared with the NC group. An i.t. administration of bvPLA2 significantly reduced the levels of IL-4, IL-5, and IL-13 in the lungs. The levels of these Th2 cytokines in the bvPLA2 treated group were similar to that of the NC group (Figure 4).

### 2.4. bvPAL2 Suppresses the Secretion of Total IgE in Serum Induced by OVA

**Figure 5:**
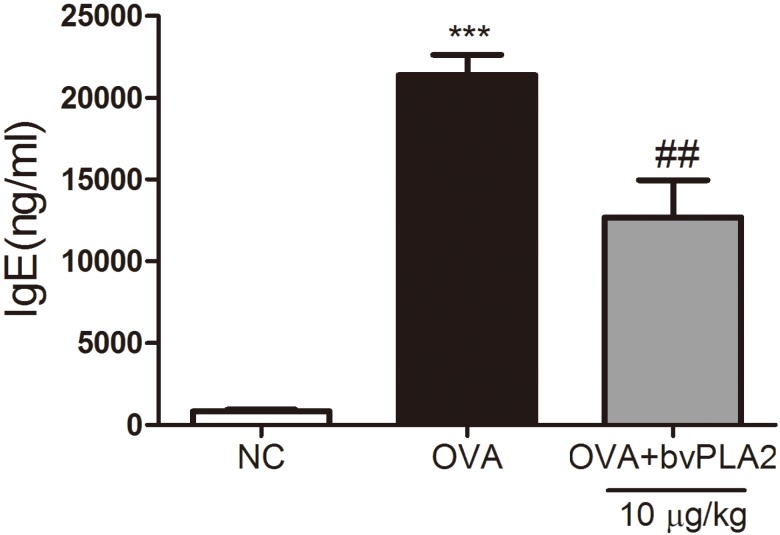
Effects of bvPLA2 on the production of total IgE in the OVA challenged mice. The total IgE levels of all mice in each group were determined by ELISA kit. Normal control mice treated with PBS alone (NC), OVA-challenged mice treated with PBS (OVA), and OVA-challenged mice treated with an i.t. injection of bvPLA2 (10 µg/kg; OVA+PLA2). The statistical analyses were conducted by one-way ANOVA followed by Newman-Keuls multiple comparison test (*** *p* < 0.001 vs. NC group and ^##^ *p* < 0.01 vs. OVA+PLA2 (10 µg/kg) group; *n* = 5).

A primary feature of the allergic asthma is an increase of blood IgE production [12]. Resultantly, the serum level of total IgE was significantly elevated in the OVA group compared with the NC group, implying that the induction of allergic airway inflammation was successful in this study. The level of total IgE was dramatically reduced in the bvPLA2 treated group compared with the OVA group (Figure 5). As for the bvPLA2, there was an inhibitory effect on the production of IgE in the OVA-challenged allergic airway inflammation mice.

### 2.5. bvPLA2 Suppresses LTB4 Production in the Lungs Induced by OVA

**Figure 6:**
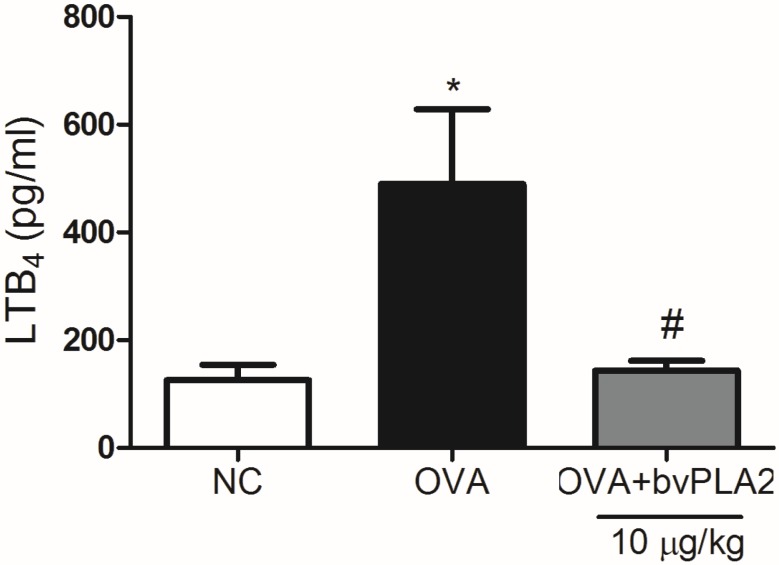
Effects of bv on the LTB4 expression on the OVA challenged mice. LTB4 protein in lung homogenate was determined via ELISA kit. Normal control mice treated with PBS alone (NC), OVA-challenged mice treated with PBS (OVA), and OVA-challenged mice treated with an i.t. injection of bvPLA2 (10 µg/kg; OVA+PLA2). The statistical analyses were conducted by one-way ANOVA followed by Newman-Keuls multiple comparison test (* *p* < 0.05 vs. NC group and ^#^ *p* < 0.05 vs. OVA+PLA2 (10 µg/kg) group; *n* = 5).

Leukotriene B4 (LTB4) is a potent inflammatory mediator for numerous cells involved in airway inflammation, including neutrophils, macrophages, eosinophils, monocytes, mast cell progenitors, and effector T cells [13]. As shown in Figure 6, bvPLA2 showed a significant reduction of LTB4 production compared with the OVA group. This result indicates that bvPLA2 inhibited chemotactic factors, such as LTB4 production in OVA induced allergic airway inflammation mice model (Figure 6).

### 2.6. bvPLA2 Alleviates Histological Change in the Lung Tissues of an OVA-Induced Airway Inflammation Model

**Figure 7:**
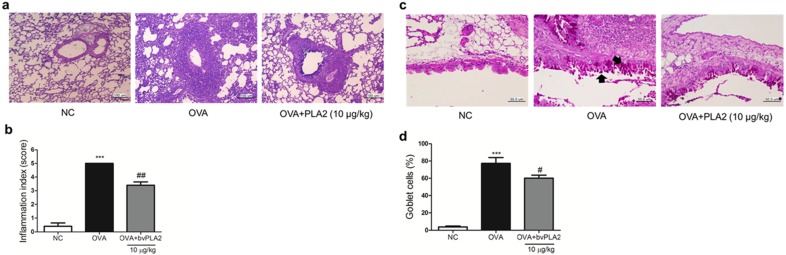
Effects of bvPLA2 on the histopathological changes in the lung tissue of OVA challenged mice. (**a**) To observe the infiltration of inflammatory cells, the lung tissues were stained with H and E (magnification 200×); (**b**) the lung inflammation severity was scored by a five-point scoring system; (**c**) lung tissues were stained with PAS (magnification 400×) as described in the Material and Methods. The black arrows indicate goblet cells; and (**d**) the percentage of goblet cells per bronchiole. Normal control mice were treated with PBS alone (NC), OVA-challenged mice were treated with PBS (OVA), and OVA-challenged mice were treated with 10 µg/kg bvPLA2 (OVA+PLA2 (10 µg/kg)). The statistical analyses were conducted by one-way ANOVA followed by Newman-Keuls multiple comparison test (*** *p* < 0.001 vs. NC group, ^##^ *p* < 0.01 and ^#^ *p* < 0.05 vs. OVA+PLA2 10 µg/kg group; *n* = 5).

To access the effects of bvPLA2 on the degree of airway inflammation or goblet cell hyperplasia, the lung tissues were stained with hematoxylin and eosin (H and E) or periodic acid-Schiff (PAS). As shown in Figure 7a,b, the OVA group showed a significant increase in the infiltration of inflammatory cells into the airways and blood vessels, but this was not observed in the NC group. The bvPLA2 treated group showed a decreased infiltration of inflammatory cells compared with the OVA group. The overproduction of goblet cell hyperplasia was noticeably detected in the bronchial airway epithelium of the OVA group compared with the bvPLA2 treated group (Figure 7c,d). These results demonstrated that bvPLA2 alleviates histological changes of the lung tissue in an OVA-induced allergic airway inflammation model.

## 3. Discussion

In a murine model of OVA-induced allergic airway inflammation, we focused on the role of an i.t. administration of bvPLA2. I.t. exposure of bvPLA2 reduced the infiltration of inflammatory cells into the lung in the OVA challenged mice. Total serum IgE and Th2 cytokines in the BAL fluid were significantly reduced in bvPLA2 treated mice compared with the OVA-induced mice. A histopathological analysis revealed that an i.t. exposure to bvPLA2 substantially inhibited eosinophil infiltration into the airway and goblet cell hyperplasia. These findings clearly show an important role for the route of administration of bvPLA2 through the decline in the OVA-induced allergic airway inflammation and AHR. The asthmatic inflammation response is divided into the early- and late-phase responses to allergen inhalation [14,15]. The early asthmatic response is characterized by an IgE-mediated decrease in bronchial airflow of the airway beginning within minutes of exposure to an allergen. Activation of mast cells is considered an early-phase asthmatic response, which involves airway smooth muscle constriction, increased mucus production, and the recruitment of inflammatory cells [16]. The late response begins 4–8 h after exposure, and the airway narrowing is associated with the migration of inflammatory cells from the bloodstream into the lung parenchyma and airway epithelium [17,18]. Furthermore, airway remodeling, such as lung hyperinflation, smooth muscle hypertrophy, epithelial cell sloughing, goblet cell hyperplasia, and subepithelial fibrosis, is a characteristic feature in chronic asthma.

Cytokines, such as IL-4, IL-5, and IL-13, are all known to be produced by CD4^+^ T helper 2 (Th2) lymphocytes and are thought to contribute to hyperresponsiveness and the hypersecretion of mucus, as well as orchestrating the recruitment of inflammatory cells [19,20]. IL-4 boosts isotype class switching in B lymphocytes, resulting in the production of allergen-specific IgE that arms mast cells and basophils that carry the high-affinity IgE receptor. IL-5 is responsible to the recruitment and activation of eosinophils in the bone marrow and tissues. IL-13 causes smooth muscle hyperreactivity, goblet cell metaplasia, mucus hypersecretion, and the alternatively activated macrophage (AAM) differentiation of monocytes. In the allergic asthma model, the Th2 cytokines, such as IL-4, IL-5, and IL-13, were clearly increased in the lung tissues. In contrast, our study demonstrated that increased AHR and the Th2 type cytokine productions were diminished by intratracheal injection of bvPLA2 in the OVA-challenged mice. In addition, our previous study demonstrated that Th2 cytokines, such as IL-4, IL-5, and IL-13, were inhibited by intraperitoneal administration of bvPLA2 in the OVA-induced airway inflammation mouse model [11]. Our results suggest that bvPLA2 plays an important role in Th2 responses during sensitization. Airway remodeling, such as goblet cell hyperplasia and subepithelial fibrosis, are key features of chronic asthma [21,22]. These histological changes in the lung were observed in the present murine model. The development of goblet cell hyperplasia and subepithelial fibrosis were prevented by the i.t. treatment with bvPLA2, suggesting that bvPLA2 produced a response to the allergen that is involved in the development of airway remodeling.

Bee venom therapy has been used for medicinal purposes, including arthritis, rheumatism, chronic recalcitrant neuralgia, and immune-related diseases [23,24]. BvPLA2 is one of the major components of bee venom [8]. Bourgeois et al. reported that bvPLA2 activates T cells via production of small neoantigens [25]. Palm et al. demonstrated that bvPLA2 induced a Th2 immune response in vivo in a manner that was dependent on its enzymatic cleavage and on the IL-33 receptor component ST2. They represented bvPLA2 as a major class of noxious allergen and used about 10-fold higher protein concentrations (2.5 mg/kg for immunization), than used in our experiments. A higher dose of bvPLA2 may induce allergic reaction in OVA-immunized mice [8]. However, we used bvPLA2 (0.25 mg/kg) as a treatment but not as a noxious allergen, and bvPLA2 treatment showed protective effects in this study.

Our previous studies reported that bee venom treatment successfully protects various immune disorders by increasing Treg populations [9,26]. Furthermore, we found that PLA2 is the active compound in bee venom capable for modulating the Treg populations, both in vitro and in vivo [9,10,11]. PLA2 from bee venom belongs to group III secreted PLA2 (sPLA2) enzymes, which play important roles in a wide range of cellular functions, including phospholipid metabolism, signal transduction, and inflammatory and immune responses [27]. Chung et al. have reported that bvPLA2 directly bound to CD206 mannose receptor on dendritic cells and induces the secretion of prostaglandin E2 (PGE2), which promotes immune tolerance through Treg differentiation via PGE2 receptor signaling and prevents the neuroinflammatory responses in a 1-methyl-4-phenyl-1,2,3,6-tetrahydropyridine (MPTP)-induced mouse model of Parkinson’s disease [9]. In addition, anti-inflammatory effects bvPLA2 was also mediated by IL-10 secretion and Treg activation in cisplatin-induced acute kidney injury model [10]. Based on these results, we supposed that bvPAL2 could inhibit inflammatory responses by modulating Treg cells through the CD206 mannose receptor. We, and other groups, have tested the necessity of enzyme activity of this enzyme for therapeutic effect on several disease model comparing naïve and heat- or chemical-inactivated bvPLA2 and reported that enzymatic activity is critical for its beneficial effects.

Leukotrienes are potent lipid mediators that are critical in the pathology of asthma, especially LTB4, which is involved in the development of severe asthma and asthma exacerbations [28]. Several groups of PLA2s have shown to be linked to LTB4 generation [29,30,31], but the relation between bvPLA2 and LTB4 production is not clearly discovered. In our experiment, LTB4 was increased in the OVA-induced airway inflammation model, as expected. However, bvPLA2 treatment suppressed the increment, suggesting that bvPLA2 can regulate the PLA2-mediated metabolic pathway in the lung. Moreover, other groups and we have tested whether enzymatic activity of bvPLA2 is necessary for its therapeutic effect using heat- or chemically-inactivated bvPLA2, and found that enzymatic activity of bvPLA2 is necessary [8,9,10]. Further studies to elucidate targets of this bee venom-derived enzyme for inhibiting lung inflammation are needed.

In the previous report, bvPLA2 had an inhibitory effect on OVA-induced allergic airway inflammation through the induction of regulatory T cells [11]. The exact mechanisms underlying the suppressive role of intratracheal administered bvPLA2 during the development of allergic asthma are yet to be defined. The drug administration during ovalbumin sensitization is not therapeutically relevant. Although, the administration timing we used has little relevance for clinical intervention, the data of our present experiment can provide further knowledge of bvPLA2 as a latent drug against lung inflammation.

## 4. Materials and Methods

### 4.1. Experimental Animals

Seven-week-old female BALB/c mice were purchased from Charles River Korea (Seungnam, Korea). The animals were housed at the Center for Laboratory Animal Care at the University of Kyung Hee. The animals were maintained under pathogen-free conditions with air conditioning, a 12-h light/dark cycle, and had free access to food and water during the experimental period. All of the experiments involving animals are reported in accordance with ARRIVE guideline for reporting experiments involving animals and Rules for Animal Care and the Guiding Principles for Experiments Using Animals by the University of Kyung Hee Animal Care, “The use of non-human primates in research” (KHUASP (SE)-12-045).

### 4.2. Reagents

Bee venom phospholipase A2 (bvPLA2; Catalogue no: P9279, Sigma-Aldrich, St. Louis, MO, USA) and ovalbumin (OVA) were purchased from Sigma-Aldrich (St. Louis, MO, USA) and were dissolved in phosphate-buffered saline (PBS).

### 4.3. Experimental Procedures

This study was performed as previously reported with minor modifications [11]. Briefly, the mice were sensitized by intraperitoneal (i.p.) injection of 100 µg OVA emulsified in 20 mg aluminum hydroxide (alum) in a 100 µL of PBS, and boosted with the identical antigen on day 14. On days 22–24 and 29–31, the mice challenged with 1% OVA in PBS intratracheally (i.t.). The sensitized mice were administered with bvPLA2 (10 µg/kg body wt) by i.t. injection two times per week for three weeks (on days 3–17). The mice were divided into three groups, with five mice in each group. In the negative control (NC) group, mice were sensitized, treated, and challenged with PBS alone. In the OVA group, the sensitized mice were challenged with OVA, and PBS was administered via i.t. as a control of bvPLA2. On day 32, the mice were analyzed using non-invasive lung function measurements (All Medicus, Seoul, Korea) to evaluate AHR. On day 33, the mice were sacrificed, and serum, bronchoalveolar lavage (BAL) fluid, and the lung tissues were collected for further analysis (Figure 1). These experiments were performed twice.

### 4.4. Assessment of Airway Hyperresponsiveness (AHR)

Airway responsiveness to methacholine was determined on day 32 in conscious, freely-moving, spontaneously-breathing mice using a whole-body barometric plethysmography chamber (All Medicus). The mice were challenged with aerosolized increasing doses (0, 25, 50, and 100 mg/mL) of methacholine generated through an ultrasonic nebulizer for 3 min. The enhanced pause (Penh) was calculated according to the manufacturer’s protocol using a software program based on the measured parameters, such as expiratory time, relaxation time, peak expiratory flow, and peak inspiratory flow according to the following formula: ((expiratory time/relaxation time − 1) × (peak expiratory flow/peak inspiratory flow)). The results are expressed as the percentage increase in Penh after challenge with each concentration of methacholine, where the baseline Penh value (after saline challenge) is expressed as 100%. Since Penh is the same breath, it is mainly independent of the functional residual capacity, tidal volume, and respiratory rate.

### 4.5. Analysis of Bronchoalveolar Lavage (BAL) Fluid

On day 33, the BAL fluid was collected by the slow infusion and extraction of 1 mL of ice-cold PBS. This procedure was repeated three times, and the lavages were pooled (mean volume, 2.0 ± mL). The recovered BAL fluid (70%–80%) was centrifuged at 1300 rpm for 10 min at 4 °C. The cell pellet was suspended with a fixed volume (1 mL per sample) of PBS and adhered to glass slides using cytocentrifugation (Sandon, Waltham, MA, USA). The total leukocyte and differential cell counts in the BAL fluid were determined by staining with Diff-Quick solution (Life Technologies, Auckland, New Zealand). A maximum of 500 cells were counted under a microscope and, based on their morphological criteria, were classified as macrophages, lymphocytes, neutrophils, or eosinophils. The results are expressed as the total cell number × 10^4^.

### 4.6. Immunoglobulin Assay

Blood was collected from the mice and was left at room temperature (RT) for 1 h and then was centrifuged for 10 min at 2500 *g* to separate the serum. The serum was diluted (1:250) with 5% FBS in PBS (assay diluent), and the total IgE levels were measured using a commercially available sandwich enzyme-linked immunoassay kit (ELISA) (BD Bioscience, San Diego, CA, USA). In brief, a 96-well immunoassay plate was coated with anti-mouse IgE monoclonal antibody (mAb) overnight at 4 °C. The coated plate was blocked with 5% FBS in PBS for 1 h at RT. Subsequently, the plates with diluted serum samples and standards were incubated at RT for 2 h. After a wash step, 100 µL of Secondary peroxidase-labeled biotinylated anti-rat IgE mAb was incubated in assay diluent for 1 h. The peroxidase reaction was initiated by the addition of 100 μL of the 3, 3’, 5, 5’-Tetramethylbensidine (TMB) substrate solution for 30 min, and then the reaction was stopped by addition of 50 μL of the TMB stop solution. The optical density was measured at 450 nm using a microplate reader (SOFT max PRO, version 3.1. software, Sunnyvale, CA, USA).

### 4.7. Th2 Cytokines Assays

The lung was homogenized in 1 mL of T-PER tissue protein extraction reagent (Thermo Scientific, Waltham, MA, USA) containing a 1 mg/mL of protease inhibitor cocktail (Roche). The lung homogenates were centrifuged at 9000× *g* for 10 min at 4 °C. The levels of the Th2 cytokines, such as IL-4, IL-5, and IL-13, were measured using a quantitative sandwich ELISA kit (BD Bioscience for IL-4, IL-5, and R and D Systems, Minneapolis, MN, for IL-13, USA). A 96-well plate (Costar, Corning, NY, USA) was coated overnight at 4 °C with anti-mouse IL-4, IL-5, or IL-13 monoclonal antibodies (mAbs) in coating buffer. After washing with 5% PBS containing 0.05% FBS in PBS, the plate was blocked with 5% FBS in PBS or 1% Bovine Serum Albumin (BSA) in PBS for 1 h at RT. Subsequently, the plate was loaded with 100 µL of the supernatants from the lung homogenates and incubated for 2 h at RT. After washing, the secondary peroxidase-labeled biotinylated anti-mouse IL-4, IL-5, or IL-13 mAbs in assay diluents were added for 1 h. Finally, the plates were treated with the TMB substrate solution (BD Bioscience, San Diego, CA, USA) for 30 min, and the reaction was stopped by the addition of 50 µL of the TMB stop solution. The optical density was measured at 450 nm using a microplate reader (SOFT max PRO, version 3.1. software, Molecular Devices, Sunnyvale, CA, USA). All of the results were normalized to the total amount of lung tissue protein in each sample. The protein concentrations were determined using the Bradford Protein Assay Kit (Bio-Rad, Hercules, CA, USA).

### 4.8. Leukotriene B4 Assay

The level of LTB4 in the lung homogenates was determined by an ELISA kit, and performed according to the manufacturer’s instruction (Enzo Life Sciences, Farmingdale, NY, USA). The detection limits for LTB4 is 11.7 pg/mL.

### 4.9. Histological Analysis

The lungs were removed from all of the experimental mice. The left lungs were fixed in 10% neutral-buffered formalin dehydrated and embedded in paraffin; then, the tissues were cut into 4-μm thick sections and stained with hematoxylin and eosin (H and E) or periodic acid-Schiff (PAS). Images of the lung tissue sections stained with H and E or PAS were acquired with an Olympus BX51 microscope (Olympus, Tokyo, Japan) equipped with a DP71 digital camera (Olympus). Airway inflammation in the H and E-stained lung sections was evaluated on a subjective score 0 to 5 on randomized, blinded sections by five independent readers according to the following criteria: 0 = normal; 1 = very mild; 2 = mild; 3 = moderate; 4 = marked; 5 = severe inflammation. The number of goblet cells within the bronchial epithelium was quantified as the percentage of PAS-positive cells. Four bronchioles randomly selected from each section of mouse lung tissue were used for the analysis, and the mean goblet cell coverage of each section was calculated.

### 4.10. Statistical Analysis

The statistical analysis of the data was conducted using the Prism 5 software (GraphPad Software, Inc., La Jolla, CA, USA). The data are presented as the means ± S.E.M. The significant differences between the means were calculated by a one-way analysis of variance (ANOVA) followed by a Newman-Keuls multiple comparison tests, as indicated in the legends. The differences were considered significant when *p* < 0.05.
